# Artificial Intelligence–based Coronary Stenosis Quantification at Coronary CT Angiography versus Quantitative Coronary Angiography

**DOI:** 10.1148/ryct.230124

**Published:** 2023-11-09

**Authors:** James Dundas, Jonathon A. Leipsic, Stephanie Sellers, Philipp Blanke, Patricia Miranda, Nicholas Ng, Sarah Mullen, David Meier, Mariama Akodad, Janarthanan Sathananthan, Carlos Collet, Bernard de Bruyne, Olivier Muller, Georgios Tzimas

**Affiliations:** From the Department of Medicine and Radiology, University of British Columbia, Vancouver, British Columbia, Canada (J.D., J.A.L., P.B., G.T.); Cardiovascular Translational Laboratory, Centre for Heart Lung Innovation & Providence Research, Vancouver, British Columbia, Canada (S.S.); HeartFlow, Mountain View, Calif (P.M., N.N., S.M.); Centre for Heart Valve Innovation, St. Paul’s Hospital, University of British Columbia, Vancouver, British Columbia, Canada (D.M., J.S.); Interventional Cardiology Department, Ramsay Générale de Santé, Institut Cardiovasculaire Paris Sud, Massy, France (M.A.); OLV Clinic, Cardiovascular Center Aalst, Aalst, Belgium (C.C., B.d.B.); and Department of Cardiology, Lausanne University Hospital and University of Lausanne, Rue du Bugnon 46, 1011 Lausanne, Switzerland (O.M., G.T.).

**Keywords:** CT Angiography, Cardiac, Coronary Arteries

## Abstract

**Purpose:**

To evaluate the performance of a new artificial intelligence (AI)–based tool by comparing the quantified stenosis severity at coronary CT angiography (CCTA) with a reference standard derived from invasive quantitative coronary angiography (QCA).

**Materials and Methods:**

This secondary, post hoc analysis included 120 participants (mean age, 59.7 years ± 10.8 [SD]; 73 [60.8%] men, 47 [39.2%] women) from three large clinical trials (AFFECTS, P3, REFINE) who underwent CCTA and invasive coronary angiography with QCA. Quantitative analysis of coronary stenosis severity at CCTA was performed using an AI-based coronary stenosis quantification (AI-CSQ) software service. Blinded comparison between QCA and AI-CSQ was measured on a per-vessel and per-patient basis.

**Results:**

The per-vessel AI-CSQ diagnostic sensitivity, specificity, accuracy, positive predictive value, and negative predictive value were 80%, 88%, 86%, 65%, and 94%, respectively, for diameter stenosis (DS) 50% or greater; and 78%, 92%, 91%, 47%, and 98%, respectively, for DS 70% or greater. The areas under the receiver operating characteristic curve (AUCs) to predict DS of 50% or greater and 70% or greater on a per-vessel basis were 0.92 (95% CI: 0.88, 0.95; *P* < .001) and 0.93 (95% CI: 0.89, 0.97; *P* < .001), respectively. The AUCs to predict DS of 50% or greater and 70% or greater on a per-patient basis were 0.93 (95% CI: 0.88, 0.97; *P* < .001) and 0.88 (95% CI: 0.81, 0.94; *P* < .001), respectively.

**Conclusion:**

AI-CSQ at CCTA demonstrated a high diagnostic performance compared with QCA both on a per-patient and per-vessel basis, with high sensitivity for stenosis detection.

**Keywords:** CT Angiography, Cardiac, Coronary Arteries

*Supplemental material is available for this article.*

Published under a CC BY 4.0 license.

## Graphical abstract

**Figure:**
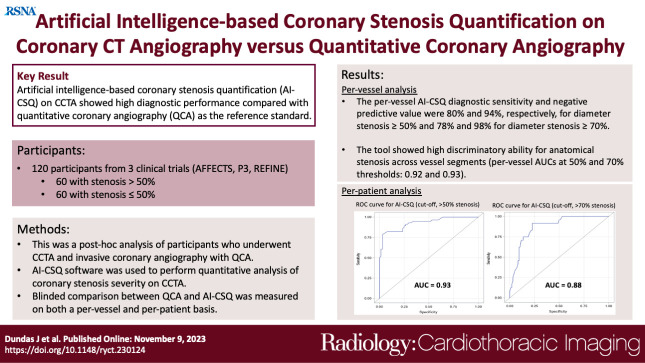

**Summary**

Artificial intelligence–based evaluation of coronary CT angiography enabled accurate identification of coronary stenosis severity, with close agreement to blinded, core laboratory–interpreted quantitative coronary angiography.

**Key Points**

■ An artificial intelligence (AI)–based coronary stenosis quantification tool displayed high diagnostic performance at both 50% and 70% stenosis thresholds, as compared with quantitative coronary angiography as the reference standard.
■ The AI-based tool exhibited high discriminatory ability for anatomic stenosis across vessel segments, including area under the receiver operating characteristic curve values of 0.92 and 0.93 at 50% and 70% thresholds, respectively.

## Introduction

Coronary CT angiography (CCTA) is an established, noninvasive technique for the diagnosis and characterization of coronary artery disease (CAD) (1). Accurate interpretation of coronary artery stenosis severity is essential to inform clinical decision-making and risk stratification (2–4). Previous studies have demonstrated high diagnostic performance of CCTA in coronary stenosis assessment when compared with quantitative coronary angiography (QCA) (5,6). CCTA diagnostic performance among less experienced readers, however, has been found to be less accurate (7,8).

The potential to develop machine learning approaches for the quantification of anatomic stenosis is of great interest to help improve interpretation efficiency and reader confidence. Therefore, we sought to evaluate the performance of a new artificial intelligence (AI)–based software service for stenosis quantification against a reference standard of invasive QCA.

## Materials and Methods

### Study Design and Sample

We conducted a retrospective, multicenter post hoc substudy including three cohorts from large clinical studies. All clinical studies were approved by the local ethics committees, and written informed consent was provided by all participants as required. The study was supported by HeartFlow, which provided the AI-based coronary stenosis quantification (AI-CSQ) software (RoadMap; HeartFlow) and performed the corresponding analysis. Employees and consultants of HeartFlow had no control of the inclusion data, information that might present a conflict of interest, or statistical analysis. The senior author had unrestricted access to the data.

**Figure 1:**
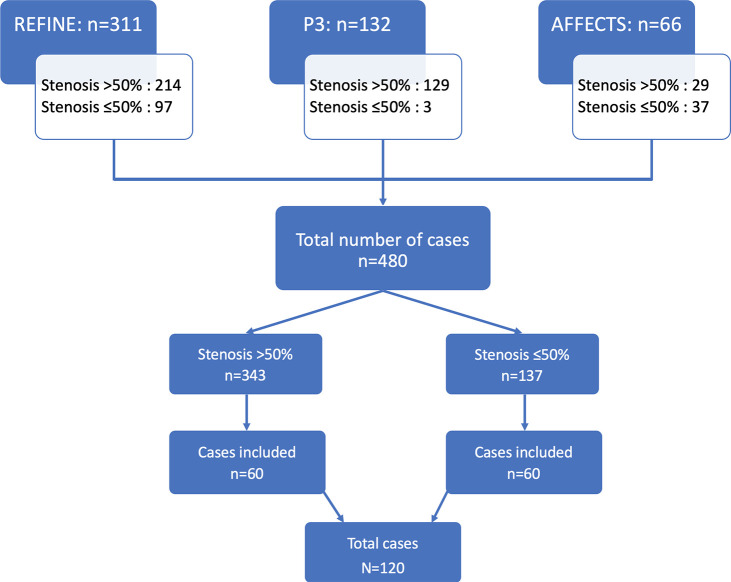
Flowchart illustrates participant selection from three international cohorts (Assessment of Fractional Flow reservE Computed Tomography Versus Single Photon Emission Computed Tomography in the Diagnosis of Hemodynamically Significant Coronary Artery Disease [AFFECTS], ClinicalTrials.gov no. NCT02973126; Precise Percutaneous Coronary Intervention Plan Study [P3], ClinicalTrials.gov no. NCT03782688; and RetrospectivE study of FFRCT compared with mFFR and cCTA In the postmarket eNvironmEnt [REFINE], unpublished data).

The study sample was derived to provide a full range of stenosis severity, with an anatomic stenosis burden similar to that observed in CCTA studies submitted for CT fractional flow reserve (CT-FFR) in clinical practice, as well as in prior accuracy studies (6,9,10). Three datasets (Assessment of Fractional Flow reservE Computed Tomography Versus Single Photon Emission Computed Tomography in the Diagnosis of Hemodynamically Significant Coronary Artery Disease, or AFFECTS, ClinicalTrials.gov no. NCT02973126 [11,12]; Precise Percutaneous Coronary Intervention Plan Study, or P3, ClinicalTrials.gov no. NCT03782688 [13,14]; and RetrospectivE study of FFRCT compared with mFFR and cCTA In the postmarket eNvironmEnt, or REFINE, unpublished data) were sorted into those with greater than 50% and those with 50% or less stenosis. We identified 343 patients with greater than 50% stenosis and 137 patients with 50% or less stenosis. A sample size of 114 patients was calculated to power 94% confidence to assess 80% sensitivity, specificity, and accuracy at the threshold of stenosis greater than 50%. A sample of 60 participants with stenosis greater than 50% and 60 participants with stenosis 50% or less were included in the final analysis (Fig 1).

All participants had symptoms suggestive of stable CAD and had undergone CCTA followed by CT-FFR and invasive coronary angiography followed by QCA. The CCTA data sets were collected retrospectively, anonymized, and exported in Digital Imaging and Communications in Medicine format for further coronary evaluation. Detection and grading of coronary stenoses at CCTA were performed using the AI-CSQ software service.

### CCTA Protocol

Participants underwent contrast-enhanced electrocardiographically gated CCTA using scanners from GE HealthCare, Siemens, Toshiba, or Philips (Table S1). Tube current, tube potential, and volume of iodine-based contrast media were adjusted based on body mass index. β-Blockers were administered if necessary, targeting a heart rate of less than 60 beats per minute. Sublingual nitrates were administered prior to scanning in all participants. Stenosis severity was graded on a per-vessel and per-patient basis.

### Invasive and Quantitative Coronary Angiography

Invasive coronary angiography was performed in agreement with clinical indications and imaging standards. All coronary arteries were defined using the Coronary Artery Disease Reporting and Data System version 2.0 (CAD-RADS 2.0) classification (15). QCA was performed by a blinded dedicated core laboratory (Cardiovascular Research Foundation) using QAngio XA 2D (version 7.3.92.0; Medis Medical Imaging) software. For each coronary artery, an end-diastolic frame was selected automatically, and the vessel was assessed in two orthogonal views. Coronary stenosis evaluation was performed using the orthogonal view with the most optimal image quality for accurately assessing the lesion. The coronary artery segments analyzed included all those with a reference diameter of 2.0 mm or greater and stenosis of 30% or greater. The reference vessel diameter (RVD) and minimal luminal diameter (MLD) were automatically calculated by the computer software. The percentage of diameter stenosis (DS) was also calculated by the software using the following formula: %DS = (1 − MLD/RVD)·100.

### AI-CSQ Protocol

**Figure 2:**
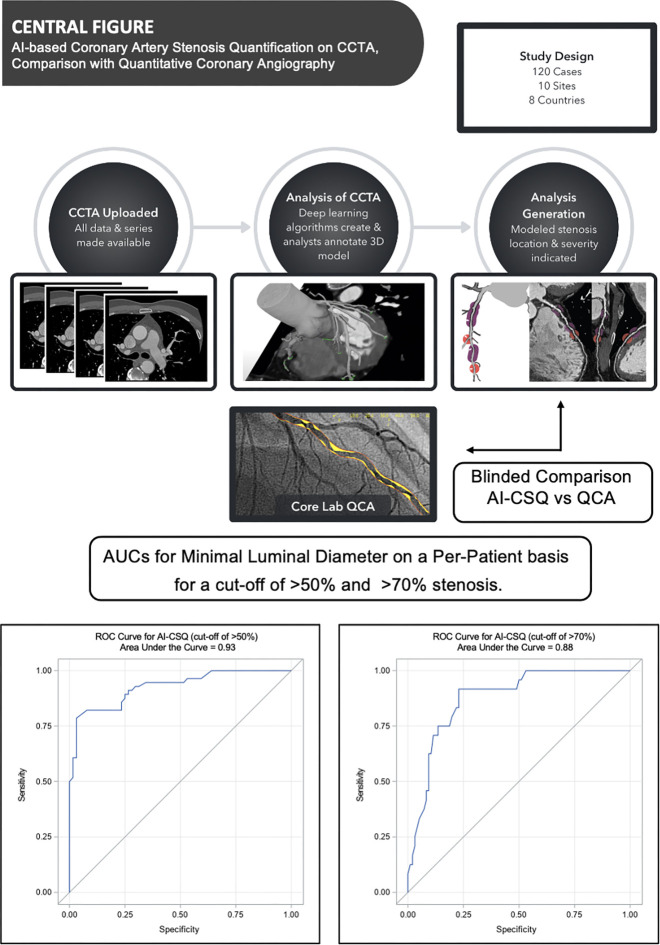
Central illustration of the study design, the process for generating artificial intelligence–based coronary artery stenosis quantification (AI-CSQ) at coronary CT angiography (CCTA), and key results. CCTA images from 120 participants were selected and uploaded in the AI-CSQ tool. An automated centerline algorithm segmented the coronary arteries (green lines), which was followed by a human quality review process, resulting in generation of a patient-specific, three-dimensional (3D) model of the arterial lumen. The tool then generated an idealized "normal" lumen for each vessel, enabling automated stenosis detection, then labeling of stenosis severity (red labels: 50%–69% stenosis; purple labels: 70%–99% stenosis) on the 3D model. We undertook a blinded comparison with invasive quantitative coronary angiography (QCA). Receiver operating characteristic (ROC) curves for the AI-CSQ tool are shown, demonstrating an area under the ROC curve (AUC) of 0.93 for detection of greater than 50% stenosis and 0.88 for detection of greater than 70% stenosis on a per-patient basis.

An AI-CSQ protocol is summarized in Figure 2. AI-CSQ relies on AI algorithms and a human quality review process for the generation of a patient-specific three-dimensional (3D) model of the arterial lumen. The analysis is based on defining the coronary vessel boundaries and subsequently extracting a 3D model of the coronary arteries that is used to perform the CSQ. The AI algorithm extracts the centerlines of main vessels, as well as side branches, while trained CT analysts review the cases to ensure all vessels are included. Extraction of the vessel centerline and lumen boundaries is performed by a U-Net–based convolutional neural network model that was trained on a proprietary database of 950 CCTA cases (about 10 million cross-sectional image samples) and 6694 CCTA cases (about 67 million cross-sectional image samples), respectively. The outer wall boundaries are extracted using the U-Net–based convolutional neural network model that was trained on a proprietary database of 2618 CCTA cases (about 26 million cross-sectional image samples). The AI-CSQ technology also includes the numerical optimization of a 3D idealized lumen model, which can be considered as a healthy reference model interpolating over the native diseased coronary lumen, thus allowing a more linear definition of reference vessel size along the centerline, including through heavily diseased segments and bifurcating lesions. Ultimately, a 3D coronary model is generated that can be analyzed at each point in a coronary artery to provide the degree of stenosis at any specific location. For coronary stenosis evaluation, the AI-CSQ software identifies the MLD and DS in vessels greater than 1.8 mm in diameter. The degree of stenosis was also assessed in the entire QCA-defined segment, which can encompass diffuse disease where multiple stenoses are present within a single QCA segment, rather than just at the location identified as the precise marked MLD. Stenoses were defined in ranges of 0%–29%, 30%–49%, 50%–69%, 70%–99%, or total occlusion. Algorithms, codes, scripts, and data are proprietary to vendors and not publicly shared as per other recently developed AI-based plaque tools.

### QCA versus AI-CSQ Comparison

Blinded comparisons between QCA and AI-CSQ software were conducted at the QCA-defined MLD and at the AI-CSQ–defined MLD within a full QCA-defined segment. Analyses were performed using cutoffs for obstructive disease of 50% or 70%. Performance of the AI-CSQ tool was assessed on a per-vessel and per-patient basis.

### Statistical Analysis

Continuous variables were summarized by reporting the means ± SDs or medians with IQRs in parentheses. Categorical variables are presented as frequencies with percentages in parentheses. The diagnostic accuracy of coronary stenosis evaluation was assessed by calculating the sensitivity, specificity, negative predictive value (NPV), positive predictive value (PPV), and diagnostic accuracy relative to the determination of stenosis of 50% or greater or 70% or greater using QCA as the reference standard on a per-patient and a per-vessel basis. The area under the receiver operating characteristic curve (AUC) was used to evaluate the diagnostic performance for 50% or greater or 70% or greater DS on a per-patient and per-vessel basis, and 95% CIs were calculated. The significance level of these AUCs was tested against the null hypothesis of no relationship better than chance between AI-CSQ and QCA (AUC = 0.5). A *P* value less than .05 was considered statistically significant. The Pearson correlation coefficient was calculated to compare the continuous measures of stenosis severity, on both a per-patient and per-vessel basis. Agreement between AI-CSQ and QCA was assessed using Bland-Altman analysis, on both a per-patient and per-vessel basis. For all per-vessel analyses, multiple vessels from the same patient were considered independently. For both per-vessel and per-patient analyses, where there were multiple lesions, the lesion with the most severe diameter stenosis (as defined by core laboratory QCA) within a vessel or patient was used. Analysis was performed using SAS software version 9.4 (SAS Institute).

## Results

### Participant Characteristics

A total of 120 participants (mean age, 59.7 years ± 10.8; 73 [60.8%] men, 47 [39.2%] women) were included in the analysis. Participant demographics and baseline characteristics are listed in Table 1. All participants had CAD as defined by QCA. At least one coronary stenosis of 50% or greater was observed in 60 participants (50%), of which 40 (66.7%) represented single-vessel disease, 18 (30%) two-vessel disease, and two (3.3%) three-vessel disease. At least one stenosis of 70% or greater was observed in 27 participants (22.5%), of which one had a total coronary occlusion (Table 2). Most participants (77.5%) had at least one lesion deemed to be functionally significant (CT-FFR ≤ 0.80), with a similar distribution of single-vessel, two-vessel, and three-vessel disease (Table 3). Stenoses of 50% or greater, defined by either QCA or AI-CSQ, were frequently functionally significant (CT-FFR ≤ 0.80 in 54 of 60 [90%] cases with QCA ≥ 50% and 69 of 83 [83.1%] cases with AI-CSQ ≥ 50%). The median calcium score was 114 Agatston units (IQR, 0–450). Table 3 also summarizes the coronary atheroma burden assessed by the coronary calcium score, stratified by severity (Agatston score < 100, 100–399, 400–999, and ≥ 1000).

**Table 1:**
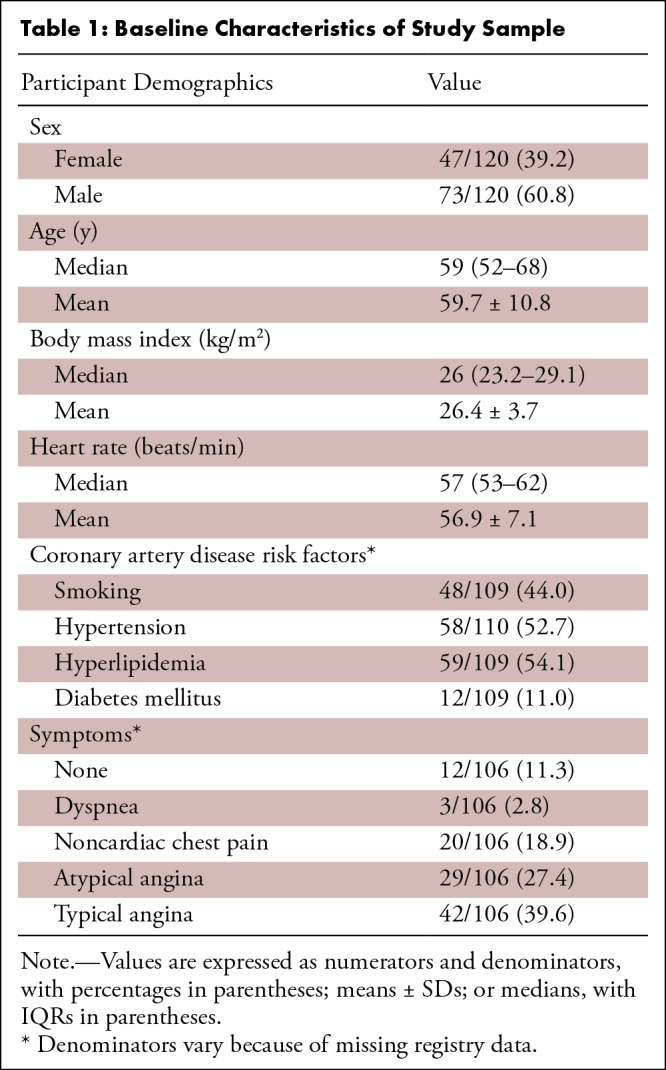
Baseline Characteristics of Study Sample

**Table 2:**
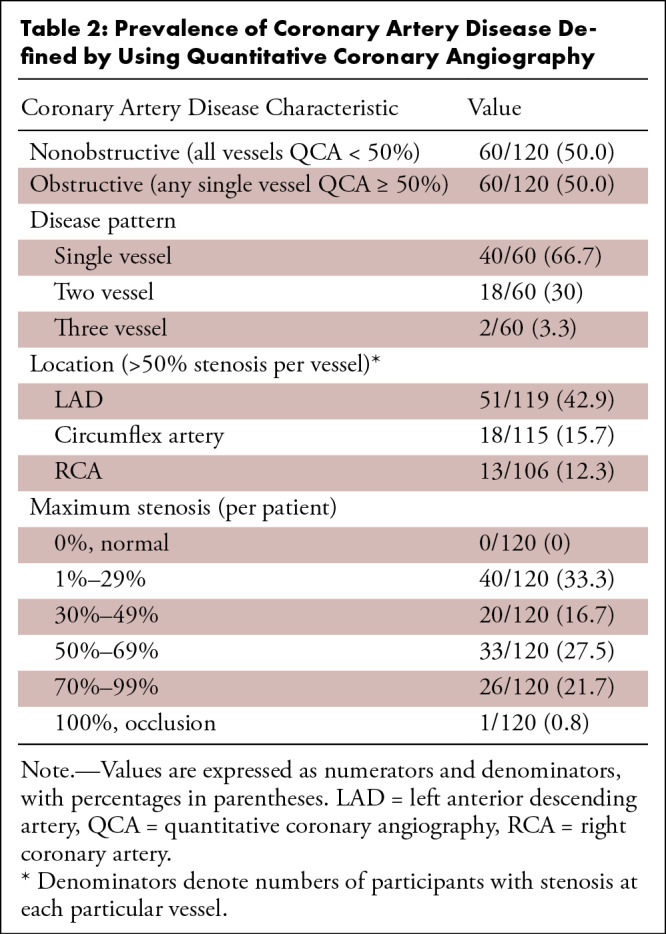
Prevalence of Coronary Artery Disease Defined by Using Quantitative Coronary Angiography

**Table 3:**
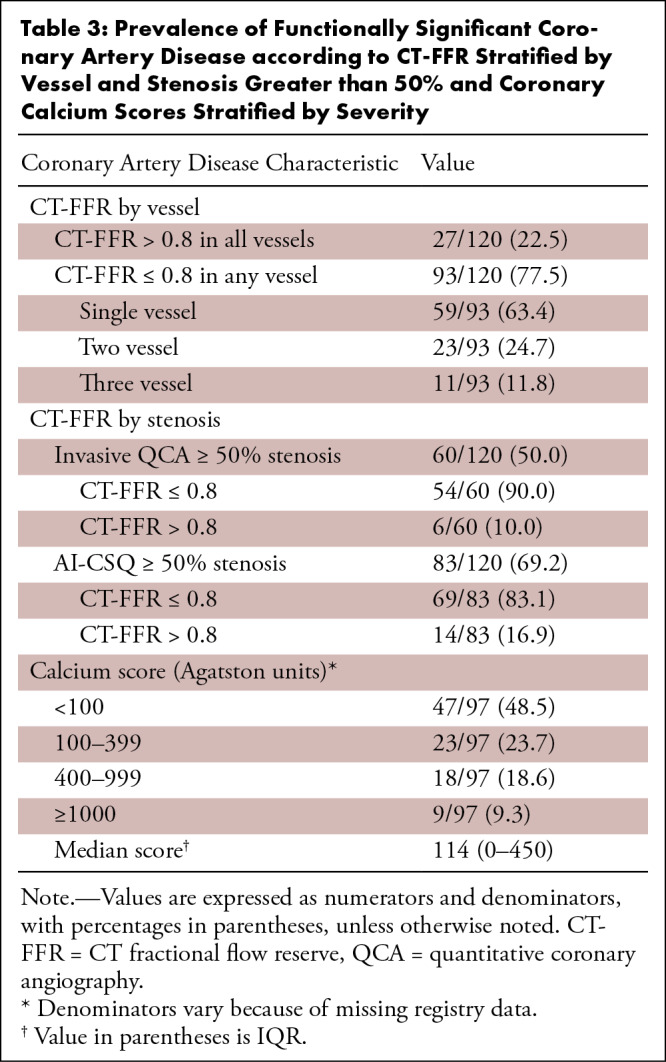
Prevalence of Functionally Significant Coronary Artery Disease according to CT-FFR Stratified by Vessel and Stenosis Greater than 50% and Coronary Calcium Scores Stratified by Severity

## Diagnostic Accuracy and Performance

### MLD Site

**Figure 3:**
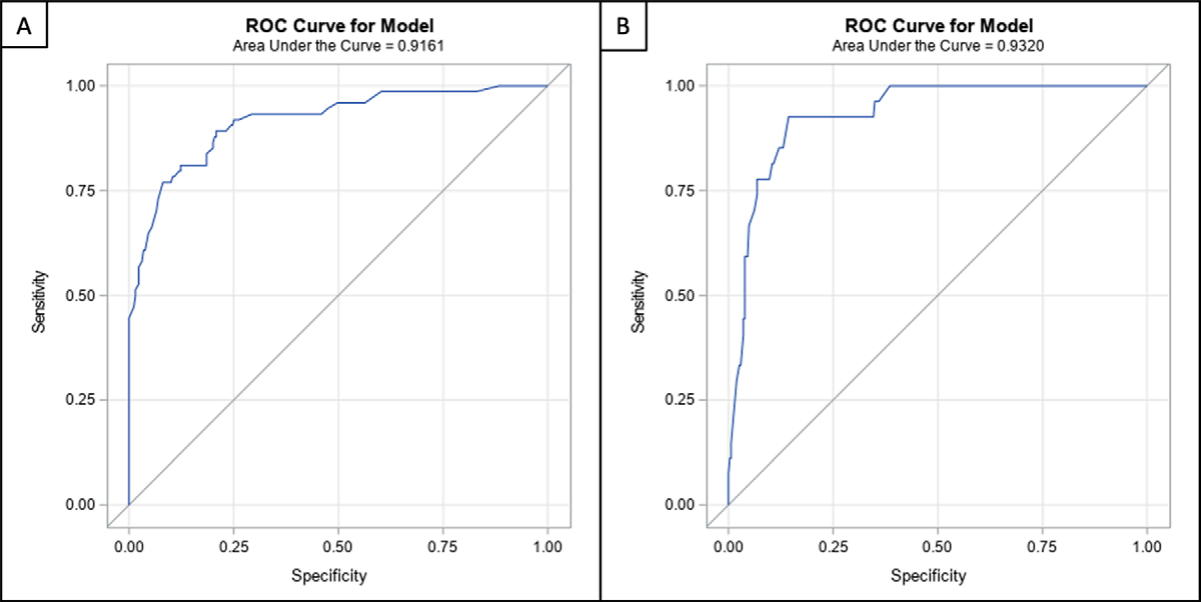
The areas under the receiver operating characteristic (ROC) curve for the model-defined minimal luminal diameter on a per-vessel basis were **(A)** 0.92 for a cutoff of 50% or greater stenosis and **(B)** 0.93 for a cutoff of 70% or greater stenosis.

**Figure 4:**
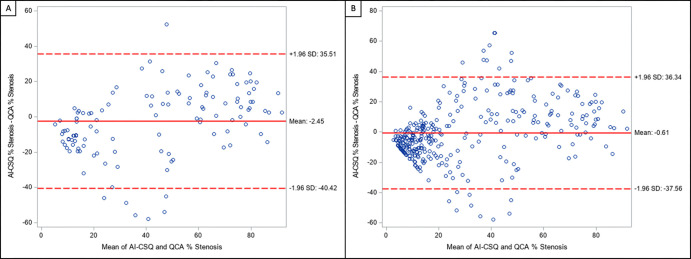
Bland-Altman analysis shows good agreement between quantitative coronary angiography (QCA) and the artificial intelligence–based coronary artery stenosis quantification (AI-CSQ) tool on both **(A)** a per-patient and **(B)** per-vessel analysis, with a mean difference of −2.45% (95% CI: −5.38%, 0.48%) and −0.61% (95% CI: −2.31%, 1.09%), respectively.

The per-patient diagnostic sensitivity, specificity, accuracy, PPV, and NPV of the AI-CSQ tool for DS of 50% or greater on a per-patient basis were 82% (46 of 56), 84% (54 of 64), 83% (100 of 120), 82% (46 of 56), and 84% (54 of 64), respectively, and 75% (18 of 24), 85% (82 of 96), 83% (100 of 120), 56% (18 of 32), and 93% (82 of 88), respectively, for DS of 70% or greater (Table 4). Similar results were found when the analysis was performed on a per-vessel basis (Table 4). The AUCs to detect a lesion with 50% or greater DS on a per-patient and per-vessel basis were 0.93 (95% CI: 0.88, 0.97; *P* < .001) and 0.92 (95% CI: 0.88, 0.95; *P* < .001), respectively. For DS of 70% or greater, the AUCs were 0.88 (95% CI: 0.81, 0.94; *P* < .001) and 0.93 (95% CI: 0.89, 0.97; *P* < .001), respectively (Figs 2, 3). The correlation coefficient of QCA and AI-CSQ for MLD was 0.79 (95% CI: 0.71, 0.85) and 0.75 (95% CI: 0.70, 0.80) (*P* < .001 for both) on per-patient and per-vessel analyses, respectively (Fig S1). The relationship between AI-CSQ and QCA for MLD of 50% or greater and 70% or greater on per-patient and per-vessel analyses is illustrated in Figure S2. Finally, there was good agreement on DS between QCA and the AI-CSQ tool on both per-patient and per-vessel analyses, with mean differences of −2.45% (95% CI: −5.38%, 0.48%) and −0.61% (95% CI: −2.31%, 1.09%), respectively, indicating small positive bias (Fig 4). Lin concordance correlation coefficients for agreement between QCA and AI-CSQ were 0.762 (95% CI: 0.676, 0.828) on a per-patient basis and 0.731 (95% CI: 0.677, 0.777) on a per-vessel basis.

**Table 4:**
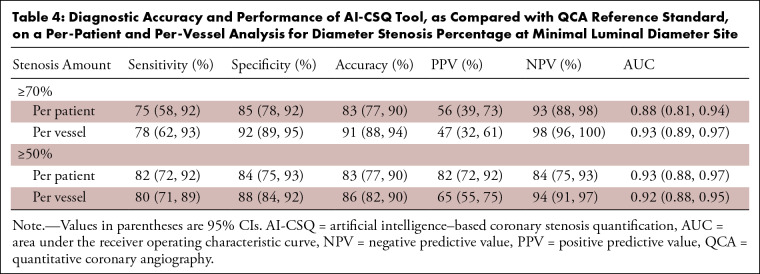
Diagnostic Accuracy and Performance of AI-CSQ Tool, as Compared with QCA Reference Standard, on a Per-Patient and Per-Vessel Analysis for Diameter Stenosis Percentage at Minimal Luminal Diameter Site

### Full QCA Segment

On a per-patient basis, the AI-CSQ tool demonstrated a sensitivity of 95% (57 of 60), specificity of 57% (34 of 60), accuracy of 76% (91 of 120), PPV of 69% (57 of 83), and NPV of 92% (34 of 37) in detecting lesions with 50% or greater stenosis. On a per-vessel basis, sensitivity was 90% (73 of 81), specificity 76% (196 of 257), accuracy 80% (269 of 338), PPV 54% (73 of 134), and NPV 96% (196 of 204) (Table S2). Using a 70% stenosis cutoff, per-patient sensitivity was 85% (23 of 27), specificity 72% (67 of 93), accuracy 75% (90 of 120), PPV 47% (23 of 49), and NPV 94% (67 of 71). On a per-vessel basis, sensitivity was 81% (26 of 32), specificity 87% (267 of 306), accuracy 87% (293 of 338), PPV 40% (26 of 65), and NPV 98% (267 of 273) (Table S2). AUC values on per-patient and per-vessel analyses were 0.86 (95% CI: 0.79, 0.92; *P* < .001) and 0.88 (95% CI: 0.84, 0.92; *P* < .001), respectively, at a 50% or greater stenosis cutoff and 0.83 (95% CI: 0.76, 0.89; *P* < .001) and 0.90 (95% CI: 0.85, 0.94; *P* < .001), respectively, at a 70% or greater stenosis cutoff (Fig S3).

### Discordant Vessels

There was a discordance in stenosis of at least 30% between the AI-CSQ tool and QCA in 36 (10.8%) of 333 vessels; discordance 30% or greater was found in 11 of 74 vessels (14.9%) with stenosis of 50% or greater defined by QCA and 18 of 91 (19.8%) vessels with stenosis of 50% or greater by AI-CSQ. Details of discordance stratified by stenosis are listed in Table 5.

**Table 5:**
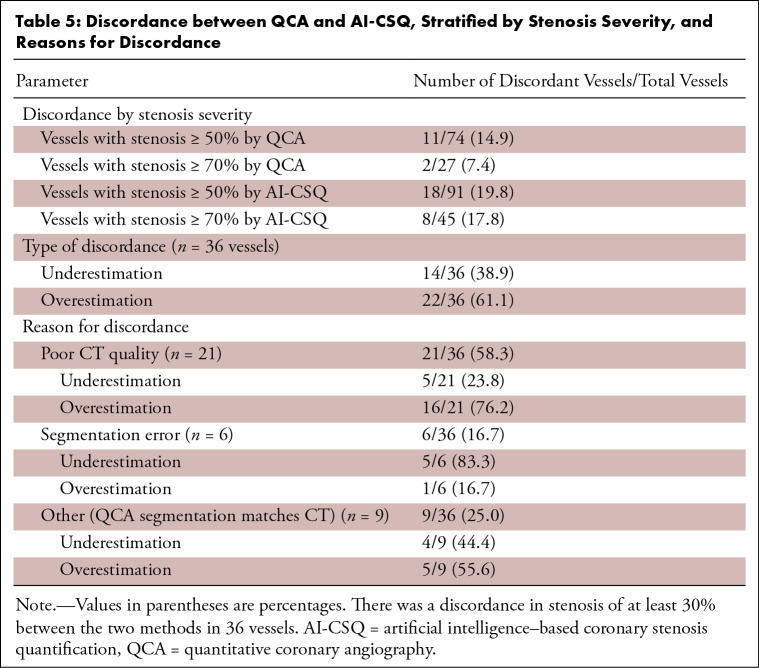
Discordance between QCA and AI-CSQ, Stratified by Stenosis Severity, and Reasons for Discordance

**Figure 5:**
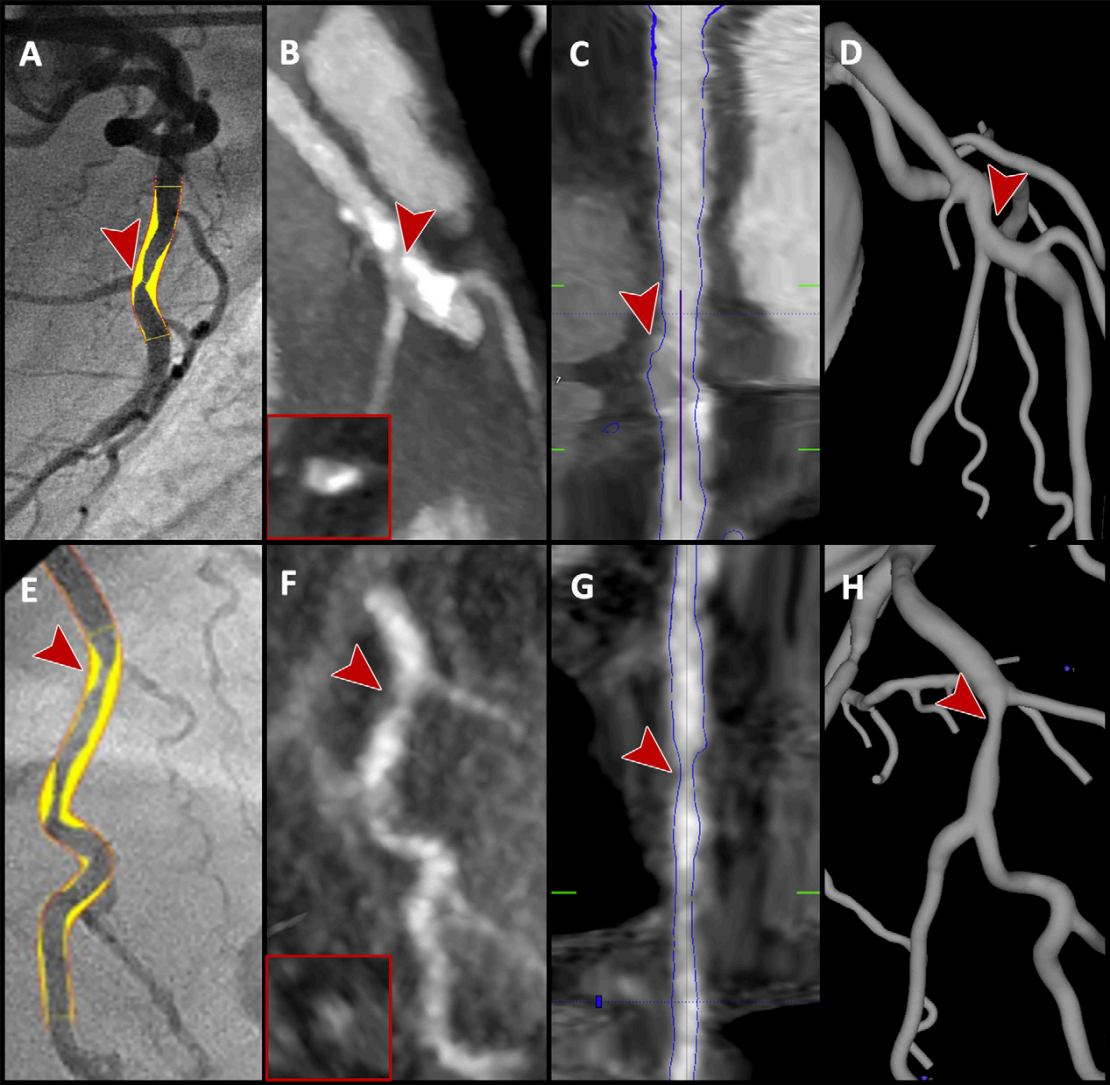
Examples of discordant cases between quantitative coronary angiography (QCA) and artificial intelligence–based coronary artery stenosis quantification (AI-CSQ). **(A–D)** Images demonstrate a vessel with a severe stenosis. **(A)** Image from invasive QCA and **(B)** CT image of the vessel, with luminal cross-section inset, indicate the location of the large side branch adjacent to the stenosis (red arrowhead). **(C)** The straightened vessel multiplanar reformat indicates the AI-CSQ determination of the lumen (blue lines), with the arrowhead demonstrating its erroneous inclusion of some of the same side branch in the main vessel lumen. **(D)** The resulting AI-CSQ three-dimensional anatomic model underestimates the severe stenosis. **(E–H)** Images demonstrate a different vessel with a severe stenosis (red arrowheads) by using **(E)** QCA, with poor image quality at **(F)** CT because of motion, leading to AI-CSQ modeling the stenosis **(G, H)** but underestimating its severity.

When discordant with the QCA reference standard, AI-CSQ demonstrated both underestimation and overestimation of luminal stenosis. Fourteen (39%) of the 36 discordant vessels were underestimates, and 22 (61%) were overestimates. Reasons for discordance were assessed and classified into three groups: poor quality CT images in 21 of 36 (58%) vessels, errors in the AI-CSQ segmentation in six of 36 (17%) vessels, and other factors (where segmentation of AI-CSQ matched QCA segmentation) in nine of 36 (25%) vessels (Table 5). Examples of discordance are shown in Figure 5.

## Discussion

In our multicenter analysis of an AI-based anatomic CT tool, using QCA as the reference standard, we found high diagnostic performance at both 50% and 70% stenosis thresholds. This observation supports the evolution of CCTA from a qualitative to an objective, quantitative tool. Importantly, from a clinical perspective, the AI-CSQ tool exhibited high discriminatory ability for anatomic stenosis across anatomic vessel segments with a high sensitivity and NPV, suggesting this tool may be helpful for readers to screen cases for anatomic stenosis. Discriminatory ability included per-vessel AUCs of 0.92 and 0.93 at 50% and 70% thresholds, respectively, a measure independent of disease prevalence, with high NPV and sensitivity on both per-patient and per-vessel levels. These findings represent what we believe are important contributions to the field and should help inform integration into clinical practice.

Germane to the present study, Griffin et al (10) used an alternative AI-based approach to evaluate coronary stenoses and reported a high diagnostic performance, with AUCs on a per-patient and per-territory level of 0.88 and 0.90, respectively, for 50% or greater stenosis and 0.92 and 0.95, respectively, for 70% or greater stenosis. Similar results were also reported in the multicenter CLARIFY trial (16) comparing an AI method with CT readers, where the sensitivity, specificity, PPV, and NPV were found to be 94.8%, 80.0%, 97.0%, 80.0%, and 97.0%, respectively, for 50% or greater stenosis and 90.9%, 99.8%, 93.3%, 99.9%, and 99.7%, respectively, for 70% or greater stenosis. These findings correlate with ours, suggesting that AI-based workflows may support more efficient, consistent, and accurate CCTA interpretations in clinical practice.

Stenosis discordance of more than 30% between the AI-CSQ tool and invasive QCA was observed in a small, yet substantial, number of cases. The proportion of discordant vessels was slightly higher in those with greater than 50% stenosis, and this was similar regardless of whether that stenosis was defined by using QCA or by AI-CSQ. While the tool more frequently overestimated than underestimated stenosis severity (61% vs 39%, respectively, of the 36 discordant vessels), clinicians using AI-CSQ should be aware of the potential for both errors, as well as the possible causes.

Poor CT image quality, including suboptimal contrast media density, streaking artifact, or misalignment (“step artifact”) accounted for the majority of errors (21 of 36, 58%), and these were predominantly overestimates (16 of 21, 76%), reinforcing the value of good quality source images and the need for a degree of caution when the AI-CSQ tool identifies potentially obstructive disease from suboptimal quality cases.

In a minority of discordant cases (six of 36, 17%), errors in segmentation predominantly caused underestimation of stenosis (five of six, 83%); this is likely related to the segmented centerline being misplaced through areas of calcified disease, which are then misinterpreted as vessel lumen. Last, in a quarter of discordant vessels (nine of 36), discrepancy between the AI-CSQ tool and QCA occurred despite adequate image quality and matching segmentation for both techniques, causing an approximately even split of over/underestimation. In these cases, discrepancies could be due to intrinsic modality differences between CT and invasive coronary angiography, as well as true anatomic differences in severity. The latter may be explained by interval disease progression between the two studies, as well as differing medical milieu, such as administration of nitrates or other antianginals. These factors likely already account for some of the known discrepancies between CT and invasive angiography in current clinical practice (17).

With the recent update to the American Heart Association/American College of Cardiology chest pain guidelines providing a class 1A designation for CCTA in both stable and acute chest pain settings (1), it is expected that the adoption and clinical integration of CCTA will continue to grow. In 2018, an analysis of the impact of implementing a CCTA pathway in the United Kingdom revealed that a higher adoption of CCTA was associated with substantial reductions in cardiovascular mortality and ischemic heart disease deaths, all without an increase in downstream testing (18). Ultimately though, the rate of adoption of CCTA will remain constrained by limited reader capacity and training. The opportunity to use an AI-based anatomic reading tool to facilitate efficient and objective anatomic interpretations across readers with varied experience is an exciting one.

The accuracy data presented herein comprise the first step in clinical validation of this technology. Importantly, our analysis is inclusive of CCTA examinations performed across multiple CT scan platforms, suggesting that this technology is viable across varied reconstruction algorithms. In addition, algorithms were developed from a large database of CT examinations and annotations that were completely distinct from those evaluated in this study, thereby avoiding potential learning bias. As noted above, we not only explored traditional per-segment anatomic accuracy versus QCA but also leveraged quantitative elements of AI-CSQ to assess accuracy and agreement between AI-CSQ and QCA at the precise MLD site. The higher sensitivity and NPV when analyzing per segment may highlight one of the key strengths of CCTA, which is the ability to anatomically exclude disease and severe stenosis. The utility of the more granular MLD stenosis evaluation is uncertain at present but should be the subject of future exploration.

Our analysis was not without limitations. Multiple vessels within the same participant were treated independently, which did not account for potentially correlated observations within the same participant. However, the similar results from the per-patient level analyses suggest this potential confounder at the per-vessel level was unlikely to have had a significant effect.

The prevalence of anatomically obstructive disease (≥50% stenosis with QCA) was 50%, which is typical of CCTA studies submitted for CT-FFR analysis. However, disease burden among individuals referred for CCTA varies greatly between institutions and is expected to be lower where CCTA is used as a rule-out test for lower-risk patients. Conversely, disease burden has shown to be increased at sites where CCTA is considered the first-line test (19). The prevalence of obstructive disease in our sample compares well to that reported in the foundational diagnostic accuracy studies validating CCTA (6,9) and AI-based stenosis assessment tools (10). Nonetheless, if we propose the potential use of AI-CSQ as an automated first-line screen for stenosis, then future studies should seek to validate it in a sample with a lower prevalence, to demonstrate applicability to the wider population seen in clinical practice.

In addition, we have not evaluated the impact of such a tool on the clinical interpretation of CCTA in practice. Additional studies should assess whether AI-CSQ can improve reader efficiency and overall accuracy and appraise any challenges that need to be overcome for readers to integrate it into their workflow. Further, this study focused exclusively on anatomic stenosis and not measures of qualitative or quantitative plaque characteristics, burden, and volume. While a limitation, an assessment of the anatomic stenosis severity is the first component of a modern CCTA interpretation according to CAD-RADS 2.0 (15), so AI-CSQ can still be of substantial assistance to the reader using the current standard of care. If quantitative plaque volume assessment techniques (20) (including AI-based analyses [21]) become more widespread in clinical practice, further work should study how these techniques interact with AI-CSQ and whether there are cumulative benefits of integrating both for prediction of patient risk and response to treatment.

Additionally, CT image quality remains important for accurate stenosis quantification by AI-CSQ, as it does in existing qualitative reporting. Large body habitus can degrade image quality further, affecting the performance of the AI-CSQ tool. The relatively low mean body mass index of 26.4 kg/m^2^ ± 3.7 in the cohort could have impacted the final results. Although image quality was identified as a factor in 58% of vessels demonstrating greater than 30% discrepancy, our analysis did not seek to correlate subjective image quality with agreement between QCA and AI-CSQ. With only 21 vessels deemed discordant because of image quality, there were too few to be further analyzed by individual cause (eg, contrast opacity, image noise, motion) or other factors (eg, scan vendor). Finally, elevated calcium scores could also potentially impact the diagnostic performance of the AI-CSQ tool. Unfortunately, our cohort size is not large enough to explore nonprespecified analyses, owing to a lack of power. Future validation studies with larger cohorts should seek to investigate the effect of both study quality and plaque characteristics, including calcium burden, on the diagnostic performance of the AI-CSQ tool to better define inherent limitations that may compromise the robustness and generalizability of the findings.

In conclusion, an AI-based CCTA stenosis adjudication tool showed high diagnostic performance compared with QCA, with high specificity for DS severity and high sensitivity for per-vessel and per-patient stenosis. The tool also demonstrated high discriminatory ability for stenosis evaluation. Further studies are needed to better understand how this tool can be integrated into clinical practice in a fashion that facilitates faster, more reproducible, and more accurate clinical CCTA reporting.
